# Protective Effects of Cannabidiol against Seizures and Neuronal Death in a Rat Model of Mesial Temporal Lobe Epilepsy

**DOI:** 10.3389/fphar.2017.00131

**Published:** 2017-03-17

**Authors:** Raquel A. Do Val-da Silva, Jose E. Peixoto-Santos, Ludmyla Kandratavicius, Jana B. De Ross, Ingrid Esteves, Bruno S. De Martinis, Marcela N. R. Alves, Renata C. Scandiuzzi, Jaime E. C. Hallak, Antonio W. Zuardi, Jose A. Crippa, Joao P. Leite

**Affiliations:** ^1^Department of Neurosciences and Behavioral Sciences, Ribeirao Preto Medical School, University of São PauloSão Paulo, Brazil; ^2^National Institute of Science and Technology for Translational Medicine, Conselho Nacional de Desenvolvimento Cientifico e TecnologicoBrasília, Brazil; ^3^Department of Chemistry, Faculty of Philosophy, Science and Languages of Ribeirao Preto, University of São PauloSão Paulo, Brazil

**Keywords:** epilepsy, cannabidiol, neuroprotection, intrahippocampal pilocarpine, animal model

## Abstract

The present study reports the behavioral, electrophysiological, and neuropathological effects of cannabidiol (CBD), a major non-psychotropic constituent of *Cannabis sativa*, in the intrahippocampal pilocarpine-induced status epilepticus (SE) rat model. CBD was administered before pilocarpine-induced SE (group SE+CBDp) or before and after SE (group SE+CBDt), and compared to rats submitted only to SE (SE group), CBD, or vehicle (VH group). Groups were evaluated during SE (behavioral and electrophysiological analysis), as well as at days one and three post-SE (exploratory activity, electrophysiological analysis, neuron density, and neuron degeneration). Compared to SE group, SE+CBD groups (SE+CBDp and SE+CBDt) had increased SE latency, diminished SE severity, increased contralateral afterdischarge latency and decreased relative powers in delta (0.5–4 Hz) and theta (4–10 Hz) bands. Only SE+CBDp had increased vertical exploratory activity 1-day post SE and decreased contralateral relative power in delta 3 days after SE, when compared to SE group. SE+CBD groups also showed decreased neurodegeneration in the hilus and CA3, and higher neuron density in granule cell layer, hilus, CA3, and CA1, when compared to SE group. Our findings demonstrate anticonvulsant and neuroprotective effects of CBD preventive treatment in the intrahippocampal pilocarpine epilepsy model, either as single or multiple administrations, reinforcing the potential role of CBD in the treatment of epileptic disorders.

## Introduction

Epilepsy is a disease characterized by spontaneous, recurrent seizures and the neurobiological, cognitive, behavioral, psychological, and social changes associated with the seizures (Fisher et al., 2014). While significant advances occurred on pharmacological treatments in the last century, up to 35% of patients with epilepsy do not respond to currently available drug treatments, especially temporal lobe epilepsy (TLE) patients with hippocampal sclerosis (Mohanraj and Brodie, 2006). TLE patients often present with severe neuron loss in CA1 and hilus subfields, poor verbal and non-verbal memory associated with the neuron loss, and neuropathological reorganization of synaptic connections within the hippocampus (Babb et al., 1991; Blumcke et al., 2013; Rodrigues et al., 2015; Peixoto-Santos et al., 2017). Additionally to the burden of seizures in the patient’s quality of life (Devinsky et al., 1995; Engel, 1996), uncontrolled seizures are known to promote neuron loss over time (Mathern et al., 2002), which could increase the cognitive deficits already seen in these patients (Rodrigues et al., 2015).

Cannabidiol (CBD), a non-psychotropic derivative of *Cannabis sativa*, is a promising candidate for the treatment of drug-resistant epilepsies. Although CBD was first isolated in 1940 and had its structure described in 1963 (Mechoulam and Hanus, 2000), the precise mechanisms of action and its interaction with the endocannabinoid system are still poorly understood. In the central nervous system, CBD is a low-affinity inverse agonist of the endocannabinoid receptor CB1 and also acts in several neurotransmitters receptors and ionic channels (Consroe et al., 1982; Pertwee, 2008; Jones et al., 2012; Hofmann and Frazier, 2013; Devinsky et al., 2014). Besides its direct effects on neurotransmission, studies in several animal models and human patients have indicated anti-inflammatory, antioxidant, anxiolytic, and neuroprotective action of CBD in the central nervous system (Hampson et al., 1998; Braida et al., 2003; Campos and Guimaraes, 2008; Jones et al., 2012). Following case reports of subjects who smoked cannabis to treat epilepsy (Consroe et al., 1975; Ellison et al., 1990), the effects of CBD on seizure control were increasingly explored. In fact, CBD’s very first observed effect in animal model was the anticonvulsant action (Carlini et al., 1973). After the initial animal observations, double-blind clinical studies showed anticonvulsant effects of CBD, with reduction of seizure frequency in patients with drug-resistant epilepsy (Cunha et al., 1980; Trembly and Sherman, 1990). More recently, CBD use as an add-on to conventional antiepileptic drug regimen was shown to reduce seizure frequency in severe drug-resistant epilepsies (Devinsky et al., 2016).

Animal models of epilepsy reproduce in great extent the symptoms and phenotypes associated with the human disease (Kandratavicius et al., 2014a). For instance, increased pre-ictal delta and theta oscillations seen in the perforant path rat model of TLE (Broggini et al., 2016) are similar to the oscillatory changes seen in TLE patients (Baulac, 2015; Malter et al., 2016). Moreover, changes in low-frequency oscillations can contribute to seizure initiation (Nazer and Dickson, 2009; Grasse et al., 2013). This similarity allowed the evaluation of the anticonvulsant effects of CBD in several animal models, such as the seizures induced by electroconvulsant currents, pentylenetetrazole-induced seizures, pilocarpine-induced seizures, and others (Karler and Turkanis, 1981; Consroe et al., 1982; Jones et al., 2009, 2012; Mao et al., 2015). For instance, CBD showed electrographic protection in electrically induced kindling and pentylenetetrazole, with decreased amplitude and duration of epileptiform local field potentials (LFPs), and increased threshold for afterdischarges (Turkanis et al., 1979; Jones et al., 2009). Additionally, studies on other nervous system disorders suggested that CBD has a neuroprotective effect (Braida et al., 2003; Mechoulam and Shohami, 2007; Hayakawa et al., 2008; Sagredo et al., 2011; Fernandez-Ruiz et al., 2013; Campos et al., 2016). However, there is still limited data regarding CBD effects on the neuropathological changes seen in the latent phase of epilepsy models.

Thus, the present study was designed to investigate the anticonvulsant and neuroprotective effects of CBD in the pilocarpine-induced status epilepticus (SE) rat model. We tested the effects of 10 mg/kg i.p. CBD before SE (CBD preventive treatment, SE+CBDp) and before and after SE (CBD preventive and therapeutic treatment, SE+CBDt) using behavioral analysis, electrophysiological analysis, and examining neuropathological hippocampal changes during SE and at short-term latent phase after SE (1 and 3 days after SE).

## Materials and Methods

### Animals

Wistar male rats (2 months old, 260–300 g; from the Central Animal Laboratory of Ribeirao Preto Medical School) were housed at room temperature (25 ± 2°C), 12:12 light/dark cycle (lights on at 7 a.m.), and free access to food and water. All procedures were performed in agreement with the ethical principles of animal experimentation adopted by the Brazilian College of Animal Experimentation (COBEA) and approved by the Ethics Committee on Animal Experiments (CETEA) of Ribeirao Preto Medical School (protocol #051/2010).

### Surgery and Treatments

Rats were anesthetized with ketamine hydrochloride 10% (0.5 ml/kg i.p. + 0.75 ml/kg i.m.; Agener, Brazil) and xylazine (0.25 ml/kg i.p. + 0.4 ml/kg i.m.; Dopaser/Calier, Spain) and body temperature was maintained constant with a heating pad (37 ± 0.5°C). The level of anesthesia was constantly checked by tail pinch reflex and, if necessary, reinforced with 10% of the initial dose. Following standard coordinates (AP = -5.6 mm, LL = ± 4.5 mm, H = -3.0 mm; Paxinos and Watson, 1997), stereotaxic surgery was performed to implant one cannula (12 mm 23G stainless steel needles; BD, Brazil) in the left dorsal hippocampus and two monopolar recording electrodes (13 mm tungsten wire coated with Teflon 60 μm diameter; AM system Inc., USA), one in the right and one in the left dorsal hippocampal hilus. A silver wire welded to a micro-screw (placed in the suture of the parietals and occipital bones) served as reference electrode. All electrodes and the cannula were fixed in the skull using dental cement. Animals were allowed to recover for 7 days before administration of pilocarpine, CBD, or vehicle.

Status epilepticus was induced by 1 μL pilocarpine hydrochloride i.h. (2.4 mg/μL diluted in 0.9 % saline; Sigma-Aldrich, USA), injected with a microsyringe (10 μL Hamilton syringe; Sigma, USA) connected to a polyethylene tube (30 cm, PE10) and gingival needle (curved 13 mm, 30G; Injex, Brazil), at 1 μL/min flow. SE was kept for 2 h, and seizure was aborted with sodium thiopental i.p. (30 mg/kg; Abbott, Brazil). CBD (99.9% pure; STI Pharmaceuticals, Brentwood, UK) was prepared 10 mg/kg diluted in 98% saline (0.15 M NaCl) and 2% Tween 80 (Sigma-Aldrich, UK) and injected i.p. in a volume of 1 mL/kg, 1 h before SE induction (groups SE+CBDp and SE+CBDt). Additionally, CBD was administered every 12 h up to euthanasia in the SE+CBDt group. Rats from all groups were euthanized at 1 or 3 days post SE.

Control groups were rats that received either one injection of vehicle i.p. (98% saline + 2% Tween 80; VH group), one injection of CBD (10 mg/kg i.p. in vehicle; CBD group), or one injection of the vehicle before SE induction (SE group). All SE animals were analyzed for SE onset latency and severity. Classification of seizure severity was according to the modified Racine’s scale: (1) mouth and facial movements, (2) head nodding, (3) forelimb clonus, (4) rearing, (5) rearing and falling (Racine, 1972).

### Gas Chromatography

The dose of CBD was chosen based on anticonvulsant effects and pharmacokinetic studies (Karler et al., 1979; Turkanis et al., 1979; Siemens et al., 1980; Consroe et al., 1982; Jones et al., 2009). Gas chromatography has used to evaluate the CBD plasma levels during the experiments and ensure CBD was present during our experiment. Blood samples were collected from rat’s tails 90 min after CBD injection for CBD, SE+CBDp, and SE+CBDt groups at day 0 (i.e., SE induction day) to assess acute CBD presence, and at days 1 and 3 for SE+CBDp and SE+CBDt. Gas chromatography evaluation followed previously established protocols for CBD detection (Karschner et al., 2010; Andrews and Paterson, 2012). CBD was detectable in the plasma at day 0 (**Supplementary Figure S1**). One and three days after administration, CBD plasma concentration was reduced or absent in SE+CBDp rats, whereas increasing levels of CBD were detected in the plasma of SE+CBDt rats (**Supplementary Figure S1**). SE+CBDt rats also had a higher variability in CBD plasma levels than SE+CBDp rats. Thus, CBD was detectable in almost all rats from the SE+CBD groups within the temporal window of our experiment.

### Electrophysiological Analysis

Spontaneous LFPs were recorded 7 days after the animal surgery. Experiments were performed in a homemade wooden box placed inside a Faraday cage, which allowed free movement of the rats (**Supplementary Figure S2**). Analogic signal were amplified, filtered (×100, 0.5–500 Hz, P55 pre-amplifier, Grass Technologies), and digitized (sampling rate of 400 Hz) utilizing an analogic-digital converter (Powerlab/16s; ADInstruments, Inc., Australia), connected to a computer, and recorded on LabChart 7.2 software (ADInstruments, Australia). Concomitant video recording was performed with a digital camera (webcam) positioned inside the wooden box (video-EEG).

LFP recording was carried out during 20 min (baseline recording or after administration CBD) followed by and additional 2-h recording after SE induction. Oscillatory activities of SE groups (SE, SE+CBDp, and SE+CBDt) were monitored 1 h per day, at day one and day three after SE. Signal artifacts (i.e., events with amplitude 2.5 times superior to the standard deviation of the segment) were removed from LFP, and data was analyzed using custom-made MATLAB scripts (The Mathwork, Natick, MA, USA). Power spectra densities (PDs) were calculated using mtspectrumc scripts from Chronux package^1^. Chronux parameters used were: *f*pass = [0 100], tapers = [3 5], err = [0 0.05], trialave = 1, pad = 0 (Lopes-Aguiar et al., 2013). In addition, relative power spectra (normalized to the PSD sum across frequencies) in delta (0.5–4 Hz) and theta (4–10 Hz) were calculated. The relative (normalized) power band estimates the spectral density at delta and theta in relation the entire spectrogram (Moshel et al., 2013). Spectral energy from 58 to 62 Hz was excluded to avoid noise contamination. Power spectra were compared among groups to evaluate the effect of CBD treatment on oscillatory activity.

### Open Field Test

Open field test was performed to evaluate the post ictal locomotion of the rats. An activity monitor apparatus (Insight, Brazil) was used to analyze total distance explored, the number of vertical activities (the number of times an animal rises in its hind limbs), time explored on the borders and center of the arena (**Supplementary Figure S3**). The testing protocol consisted of placing the animal in the center of the box (habituation) for 3 min and then test for 8 min, according to previously tested protocols (Wolf et al., 2016). After each test, open field was cleaned with acetic acid (0.1% v/v). All data were automatically collected via software (Insight, Brazil).

### Histological Evaluation

One or three days after SE, rats were anesthetized with sodium pentobarbital (80 mg/kg i.p.) and perfused intracardially with 100 mL phosphate buffered saline (PBS, 0.1 M pH 7.4), followed by 250 mL 4% (w/v) paraformaldehyde in PBS. Brains were then removed from skull, post-fixed by immersion in 4% paraformaldehyde-PBS for 4 h, dehydrated and embedded in paraffin. Eight-micrometer-thick coronal sections (-3.3 to -3.6 mm from bregma) were mounted on slides and submitted to Fluoro-Jade B (FJB) histochemistry and NeuN immunohistochemistry for evaluation of neurons in degeneration and neuronal density, respectively.

Fluoro-Jade B histochemistry followed published protocol (Schmued et al., 1997; Do Val-da Silva et al., 2016). Brains were deparaffinized, hydrated, and immersed in the following sequence: NaOH 1% (w/v) solution in 80% (v/v) ethanol (5 min); 70% (v/v) ethanol (2 min); distilled water (2 min); potassium permanganate 0.06% (w/v) (15 min); two baths of distilled water (1 min); 0.01% Fluoro-Jade B (Histo-Chem Inc., USA) in 0.01% acetic acid (30 min); and three baths of distilled water (1 min). Sections were dehydrated and mounted with Krystalon (Harleco/EMD Millipore, USA).

Adjacent coronal sections were submitted to immunohistochemistry for the detection of NeuN epitope with an anti-mouse antibody (code MAB377, Chemicon/EMD Millipore, USA), following previously published protocols (Peixoto-Santos et al., 2012; Kandratavicius et al., 2014b). Briefly, sections were submitted to endogenous peroxidase block, microwave antigenic retrieval with 50 mM Tris-HCl pH 9.6, and overnight NeuN antibody solution in skim milk blocking buffer at 1:1,000 dilution. Primary antibody detection was done with rabbit anti-mouse IgG (E0354, Dako, Denmark) in blocking buffer (1:200), avidin-biotin-peroxidase kit (Elite ABC kit, Vector, USA) amplification, and diaminobenzidine (DAB, Pierce/ThermoFisher, USA) as a chromogen. Sections were dehydrated and mounted with Krystalon (Harleco, USA).

Micrographs from hippocampal subfields were collected with AxioVision software and MR AxioCam 15MP coupled to an Axioscope A1 microscope (Zeiss, Germany). FJB positive (FJB+) cells were counted in 30 squares of 2,500 μm^2^ in the contralateral and ipsilateral hilus, CA3, and CA1 subfields. NeuN positive cells were counted in 12 squares of 2,500 μm^2^ in granule cell layer, 6 squares of 2,500 μm^2^ in the hilus, and 10 squares of 2,500 μm2 in CA3 and CA1 subfields, and neuron density was estimated with Abercrombie correction, as described elsewhere (Do Val-da Silva et al., 2016).

### Statistical Analysis

Student’s *t*-test or ANOVA followed by the Sidak *post hoc* test were used for parametric variables, whereas non-parametric variables were evaluated with Mann–Whitney’s. For analysis over time between different groups, we used two-way ANOVA for repeated measures mixed model (RM ANOVA) followed by a Sidak *post hoc* test. In the results section, the mention of Sidak *post hoc* test was omitted, but this *post hoc* test was applied to all ANOVA tests and is mentioned in the figure captions. All statistical procedure was performed using SPSS 17. Statistical significance was considered at *p* < 0.05.

## Results

### Behavioral Effects of CBD

**FIGURE 1:**
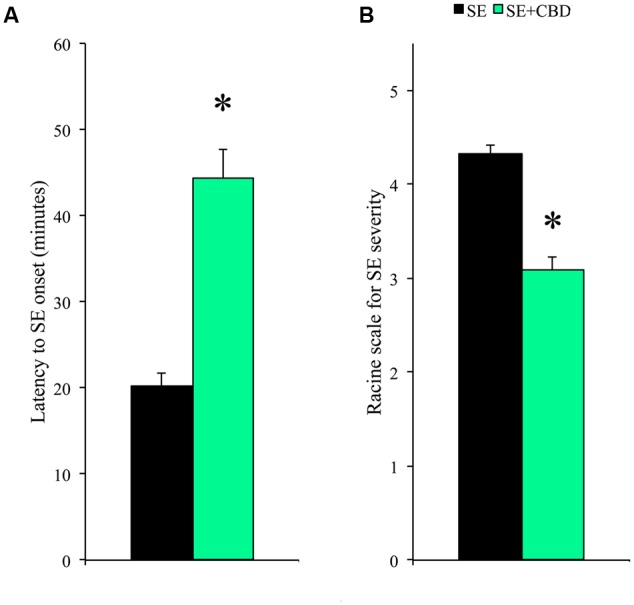
**Cannabidiol (CBD) modulation of SE latency and severity.** Rats submitted to CBD before SE induction (SE+CBD, green bar) had increased latency to SE onset **(A)** and decreased SE severity **(B)**, compared to rats not injected with CBD (SE group, black bar). The ^∗^ indicates difference from SE group, and data are expressed as mean ± standard error.

As expected, VH and CBD groups showed no behavioral change. A higher number of SE+CBD rats did not developed SE when compared to SE group (35.1% vs. 15.9%; χ^2^, *p* = 0.025). The SE+CBD groups, when compared to SE group, had increased latency to SE (44 ± 3 min vs. 20 ± 2 min; *t*-test, *p* < 0.001; **Figure 1A**) and lower SE severity (Racine 3 vs. Racine 4; Mann–Whitney, *p* < 0.001; **Figure 1B**). Moreover, none of the SE+CBD rats presented Racine 5 seizures or died, whereas 36.1% of rats from SE group had Racine 5 seizures and one rat died during SE.

**FIGURE 2:**
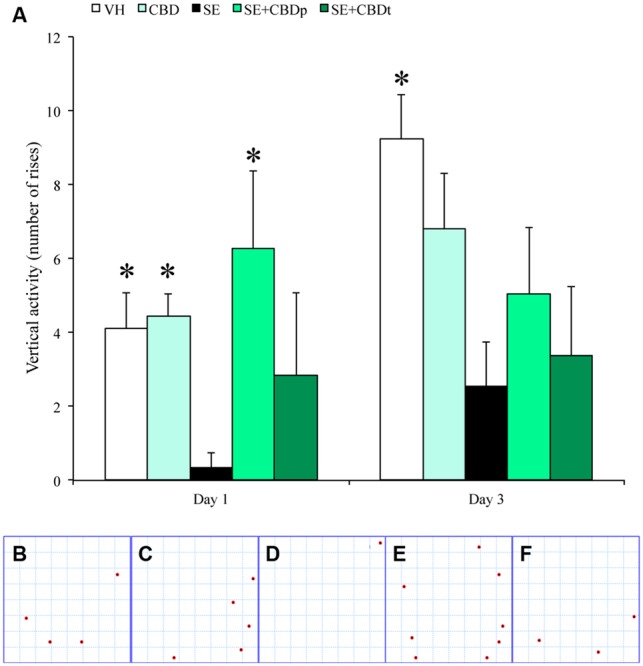
**CBD action on vertical activity of rats submitted to pilocarpine-induced SE.** One day after pilocarpine-induced SE, SE rats had a reduced vertical exploratory activity (measured as number of rises in the rat’s hind limbs), when compared to the controls (VH and CBD), and with SE rats pretreated with CBD (SE+CBDp). Representative images from the activity monitor showing the location of each rise from a VH rat **(B)**, a CBD rat **(C)**, a SE rat **(D)**, a SE+CBDp rat **(E)**, and a SE+CBDt rat **(F)**. The red dots in **B–F** indicate the places within the activity monitor where the rats rose on its hind limbs. Three days after SE, SE rats had low vertical exploratory activity only when compared to VH group **(A)**. The ^∗^ indicates difference from SE group and data are expressed as mean ± standard error.

Locomotory activity was assessed to evaluate post-ictal changes (i.e., post ictal lethargy) in rats submitted to pilocarpine-induced SE. Compared to controls (VH and CBD) and SE+CBDp, SE rats had less vertical exploratory behavior 1 day after SE (ANOVA; *p* ≤ 0.02; **Figures 2A–F**), whereas at day 3 SE had lower vertical exploratory activity than VH group (ANOVA, *p* = 0.008; **Figure 2A**). There was no difference between groups regarding total exploratory behavior or time spent on the arena borders vs. arena center (data not shown). Therefore, CBD treatment modulated the behavioral features of pilocarpine-induced SE and normalized the post-SE locomotory activity in the SE+CBDp group shortly after SE.

### Electrophysiological Modulations of CBD

**FIGURE 3:**
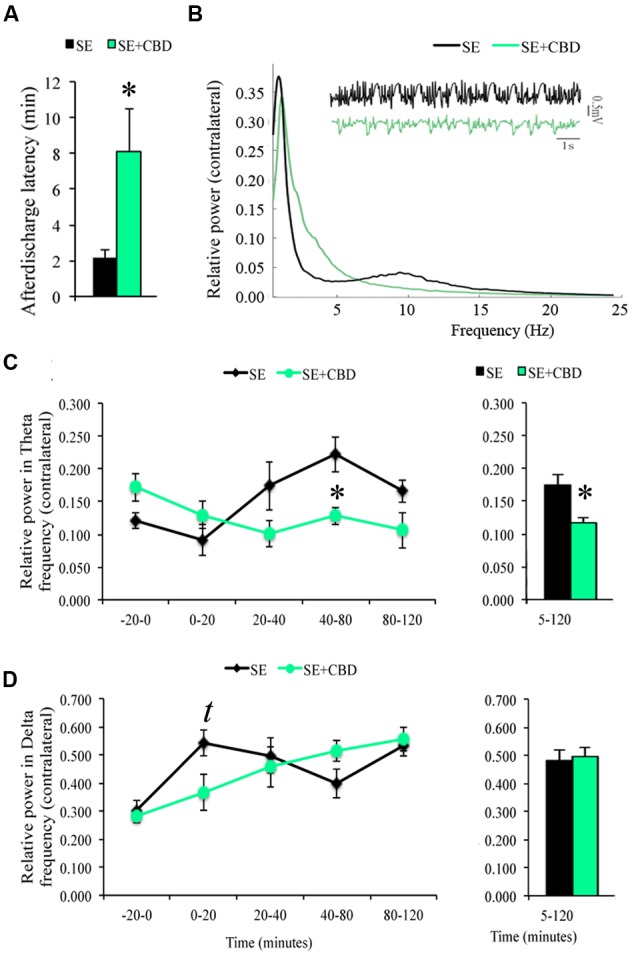
**Cannabidiol effects on spontaneous local field potential (LFP) of the contralateral hippocampus during SE.** CBD injection prior to SE (SE+CBD group, green bars) increased afterdischarge latency **(A)**, compared to untreated rats (SE group, black bars). Relative power of frequencies 0.5–25 Hz from a representative SE rat (black line) and a SE+CBD rat (green line) and a raw LFP hippocampal recording from the same examples **(B)**. Evaluating the LFP recording in time blocks, SE+CBD had a lower relative power in theta between 40 and 80 min and in the whole SE **(C)**. As for delta oscillation, SE+CBD had a trend toward lower relative power in delta in the first 20 min of SE but no difference in the whole SE recording **(D)**. The ^∗^ indicates difference from SE group, *t* indicates trend toward difference, and data are expressed as mean ± standard error.

The LFP recording of animals during SE showed increased time to contralateral epileptiform afterdischarges onset in SE+CBD groups compared to SE group (8.09 ± 2.42 min vs. 2.10 ± 0.52 min; *t*-test, *p* = 0.04; **Figure 3A**). There was no difference regarding afterdischarge onset ipsilateral to the pilocarpine microinjection (7.63 ± 2.52 min vs. 2.36 ± 0.46 min; *t*-test, *p* = 0.06). A qualitative evaluation of the raw LFP recording as well as the whole frequencies from 0.5–25 Hz shows differences in electrographic activity between the SE and the SE+CBD groups (**Figure 3B**). Amongst the frequencies in the 0.5–25 Hz interval, increased hippocampal delta (0.5–4 Hz) and theta (4–10 Hz) frequencies are hallmarks of ictal electrographic activity, as mentioned above. Thus, ictal powers of these low-frequency oscillations are relevant to understand the neuroprotective actions of CBD during seizures and the post-ictal electrographic changes in the pilocarpine-induced SE rat model. SE+CBD groups had lower relative power in theta frequency contralateral to pilocarpine microinjection, when compared to SE group, at 40–80 min (0.12 ± 0.013 vs. 0.22 ± 0.02; RM ANOVA, *p* = 0.01) and in the full block of time (0.11 ± 0.009 vs. 0.17 ± 0.01; RM ANOVA, *p* = 0.016; **Figure 3C**). Contralateral delta band oscillation changed over time (RM ANOVA, *p* < 0.001), with a trend toward difference between SE+CBD and SE groups at 0–20 min (0.36 ± 0.06 vs. 0.54 ± 0.04) but no difference in the full block of time (**Figure 3D**). There was no difference in relative powers in delta and theta frequencies in the hippocampus ipsilateral to pilocarpine injection, when comparing the SE+CBD and SE groups (RM ANOVA; data not shown).

**FIGURE 4:**
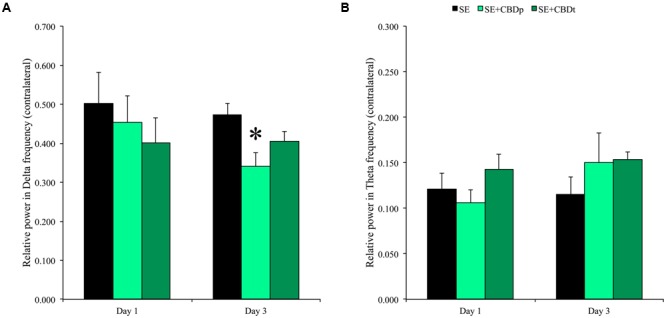
**Relative power in delta and theta frequencies 1 and 3 days after SE induction.** CBD pretreatment (SE+CBDp, medium green bars) reduced the relative power in delta, compared to SE group 3 days after SE (black bar) (**A**; RM ANOVA followed by Sidak *post hoc* test). There was no difference between SE, SE+CBDp, and SE+CBDt in delta frequency 1 day post-SE and in theta frequency 1 or 3 days post-SE **(B)**. The ^∗^ indicates difference from SE group and data are expressed as mean ± standard error.

The evaluation of spectral power 1 and 3 days after SE showed continuous drop in relative power of delta frequencies of the SE+CBD groups, with more prominent drop in the SE+CBDp group (compare **Figure 3D** full block of time with **Figure 4A**), whereas the relative power in theta frequency of SE group dropped over time while the theta frequencies of SE+CBDp and SE+CBDt groups increased (compare **Figure 3C** and **Figure 4B**). At day one, there was no difference between SE, SE+CBDp, and SE+CBDt regarding contralateral relative power in delta (**Figure 4A**) or theta frequencies (**Figure 4B**). At 3 days post-SE, SE+CBDp had lower contralateral relative power in delta band compared to SE group (0.34 ± 0.03 vs. 0.47 ± 0.02; RM ANOVA, *p* = 0.02; **Figure 4A**). There was no difference between SE+CBDp, SE+CBDt, and SE in the theta frequency on day three (**Figure 4B**). There was also no difference in the ipsilateral delta and theta frequencies between the groups at 1 or 3 days (RM ANOVA, data now shown). CBD and VH groups had no difference in baseline or in delta and theta frequencies (data not shown). In summary, CBD increased afterdischarge latency and prevented some of the theta and delta changes seen in the pilocarpine-induced SE.

### Neuroprotective Effects of CBD in the Hippocampus

**FIGURE 5:**
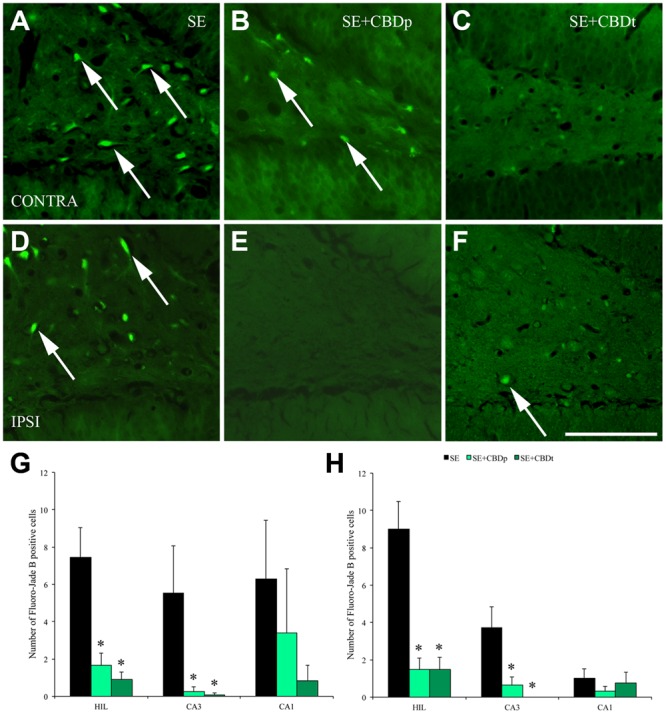
**Protective action of CBD over neuronal degeneration 1 day after pilocarpine-induced SE, evaluated by Fluoro-Jade B (FJB) staining. (A–F)**, representative micrographs from the contralateral **(A–C)** and ipsilateral **(D–F)** hilus of a SE rat **(A,D)**, SE+CBDp rat **(B,E)**, and a SE+CBDt rat **(C,F)**. Whereas SE rats present higher number of FJB positive neurons in both hilus (arrows in **A,D)**, CBD treated rats had only a few FJB positive neurons **(B,C,E,F)**. Both the contralateral **(G)** and ipsilateral **(H)** hippocampus of SE+CBDp (medium green bars) and SE+CBDt (dark green bars) had a lower number of FJB positive neurons in the hilus and CA3, compared to SE group (black bars; ANOVA followed by Sidak *post hoc* test). The white bar in **F** indicates 100 μm and the ^∗^ indicates differences from SE group. Data were presented as mean ± standard error.

**FIGURE 6:**
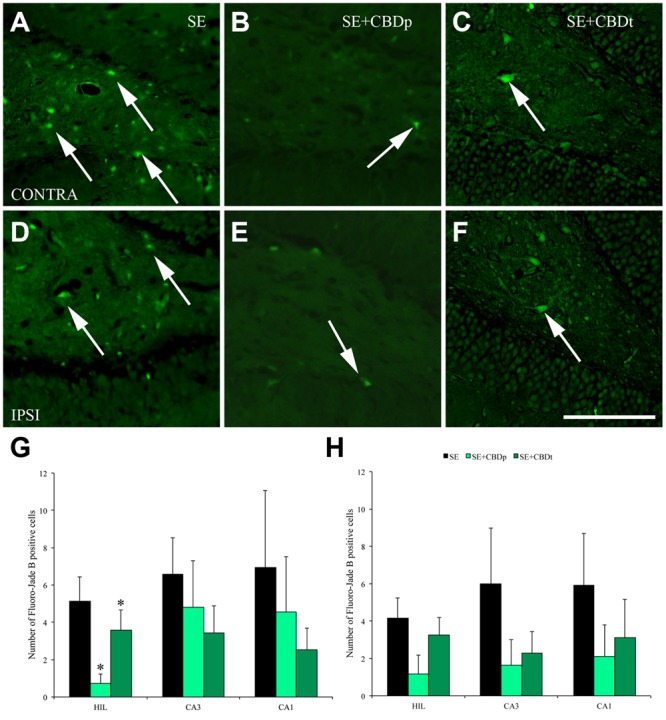
**Protective action of CBD over neuronal degeneration 3 days after pilocarpine-induced SE, evaluated by Fluoro-Jade B** (FJB) staining. **(A–F)**, representative micrographs from the contralateral **(A–C)** and ipsilateral **(D–F)** hilus of a SE rat **(A,D)**, SE+CBDp rat **(B,E)**, and a SE+CBDt rat **(C,F)**. Similar to 1 day evaluation, SE rats presented higher number of FJB positive neurons in both hilus (arrows in **A,D**), compared to CBD-treated rats **(B,C,E,F)**. SE+CBDp (medium green bars) and SE+CBDt (dark green bars) had a lower number of FJB positive neurons in the contralateral hilus **(G)**, compared to SE group (black bars), with no difference in CA3 and CA1 contralateral **(G)**, or in the ipsilateral hippocampus (**H**; ANOVA followed by Sidak *post hoc* test). The white bar in F indicates 100 μm and the ^∗^ indicates differences from SE group. Data were presented as mean ± standard error.

Acutely after SE, neurons in the hippocampus often enter a process of degeneration that leads to neuron loss over time. In our study, neurodegeneration was measured by presence and number of Fluoro-Jade B positive (FJB+) cells. No FJB+ cells were seen in the control groups VH and CBD, 1 or 3 days after vehicle or CBD injection. SE group had a high number of FJB+ cells in the contralateral and ipsilateral hilus, CA3, and CA1 at 1 (**Figure 5**) or 3 days (**Figure 6**) post SE induction. CBD injection significantly reduced SE-induced neurodegeneration, as can be visually seen in the comparison of SE+CBDp (B,E) and SE+CBDt (C,F) with SE hilus (A,D) of **Figures 5**, **6**. In agreement with the visual evaluation, SE+CBDp and SE+CBDt had significantly lower FJB+ cells in the hilus (ANOVA, *p* ≤ 0.001) and CA3 (ANOVA, *p* ≤ 0.03) of the ipsilateral and contralateral hippocampus, compared to SE group, 1 day after SE (**Figures 5G,H**). Three days after pilocarpine-induced SE (**Figures 6G,H**), SE+CBDp and SE+CBDt groups had lower FJB+ cells only in the contralateral hilus (ANOVA, *p* ≤ 0.014), compared to SE group. All SE groups (SE, SE+CBDp, and SE+CBDt) had FJB+ cells in ipsilateral or contralateral CA1 subfield at 1 and 3 days after SE, with no statistical difference between groups. Thus, CBD reduced the neurodegeneration common to the pilocarpine-induced SE rat model.

**FIGURE 7:**
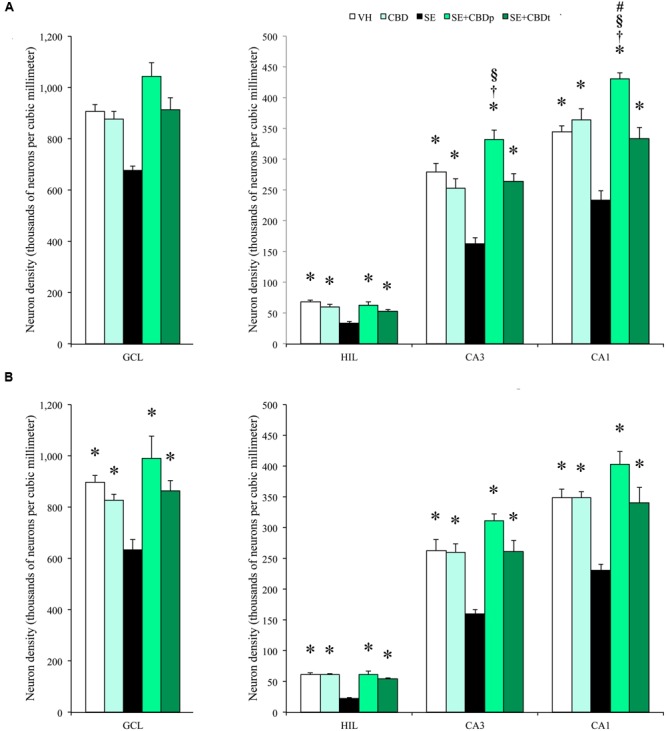
**Cannabidiol effects on hippocampal neuronal survival, 1 day after SE.** Neuronal density, estimated in contralateral **(A)** and ipsilateral **(B)** hippocampal sections from VH (white bars), CBD (light green bars), SE (black bars), SE+CBDp (medium green bars), and SE+CBDt (dark green bars) groups. All groups had higher neuron density than SE group in the hilus, CA3, and CA1 subfields of contralateral **(A)** and ipsilateral **(B)** hippocampus (ANOVA followed by Sidak *post hoc* test). In the contralateral hippocampus, SE+CBDp group had higher neuron density than CBD and SE+CBDt in CA3 and CA1. SE+CBDp had also higher neuron density than VH group in the contralateral CA1 subfield. The groups VH, CBD, SE+CBDp, and SE+CBDt had also higher neuron density than SE group in the ipsilateral granule cell layer. GCL, granule cell layer; HIL, hilus. The ^∗^ indicates differences from SE group, the ^†^ indicates difference from SE+CBDt group, the ^§^ indicates difference from CBD group, and the ^#^ indicates difference from VH group. Data were presented as mean ± standard error.

To evaluate neuron loss following SE, we analyzed neuron density, measured as NeuN positive cell density, at 1 and 3 days post-SE. One day after SE induction, SE group had lower neuron density than the controls (VH and CBD) in the contralateral (**Figure 7A**) and ipsilateral (**Figure 7B**) hilus, CA3, and CA1 (ANOVA, *p* < 0.001), as well as in the ipsilateral granule cell layer (ANOVA, *p* < 0.001). CBD injection prevented SE-induced neuron loss, as SE+CBDp, and SE+CBDt groups had higher neuron density than SE group in contralateral (**Figure 7A**) and ipsilateral (**Figure 7B**) hilus, CA3, and CA1 (ANOVA, *p* < 0.001), and in ipsilateral granule cell layer (ANOVA, *p* < 0.001; **Figure 7B**). Moreover, SE+CBDp and SE+CBDt groups had similar neuron density than the control groups in almost all hippocampal subfields evaluated. Only in contralateral CA3 and CA1, SE+CBDp group had higher neuron density than CBD and SE+CBDt (ANOVA, *p* < 0.006), and than VH, CBD, and SE+CBDt (ANOVA, *p* < 0.03; **Figure 7A**), respectively. There was no difference between groups in contralateral granule cell layer.

**FIGURE 8:**
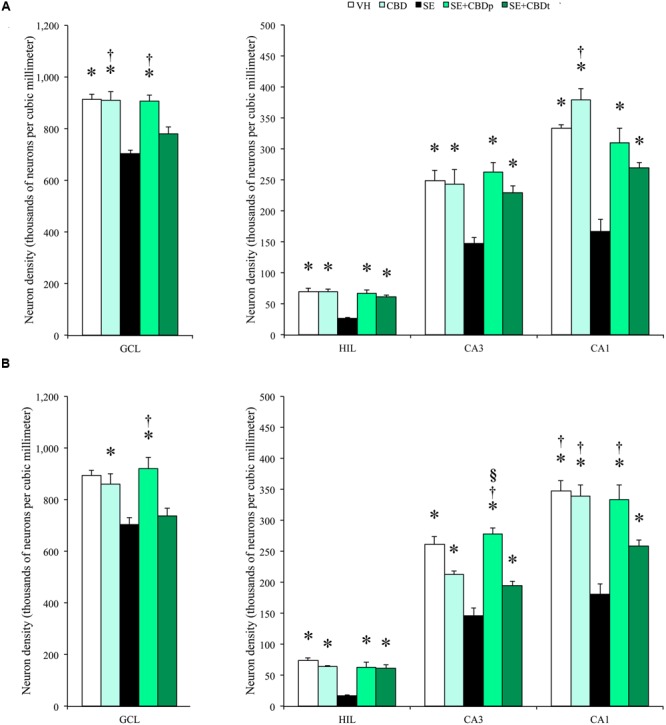
**Cannabidiol effects on hippocampal neuronal survival, 3 days after SE.** Neuronal density, estimated in contralateral **(A)** and ipsilateral **(B)** hippocampal sections from VH (white bars), CBD (light green bars), SE (black bars), SE+CBDp (medium green bars), and SE+CBDt (dark green bars) groups. The groups VH, CBD, SE+CBDp, and SE+CBDt had higher neuron density than SE group in contralateral **(A)** and ipsilateral **(B)** hilus, CA3, and CA1 subfields (ANOVA followed by Sidak *post hoc* test). In the contralateral granule cell layer, the groups VH, CBD, and SE+CBDp had higher neuron density than SE, and the groups CBD and SE+CBDp had higher neuron density than SE+CBDt. CBD group had also higher neuron density than SE+CBDt in contralateral CA1. In the ipsilateral granule cell layer **(B)**, the group CBD had higher neuron density than SE, and the group SE+CBDp had higher neuron density than SE and SE+CBDt groups. In CA3, SE+CBDp group had higher neuron density than CBD and SE+CBDt groups, and in CA1 the groups VH, CBD, and SE+CBDp had higher neuron density than SE+CBDt group. GCL, granule cell layer; HIL, hilus. The ^∗^ indicates differences from SE group, the ^§^ indicates difference from CBD group, the ^†^ indicates difference from SE+CBDt group. Data were presented as mean ± standard error.

**FIGURE 9:**
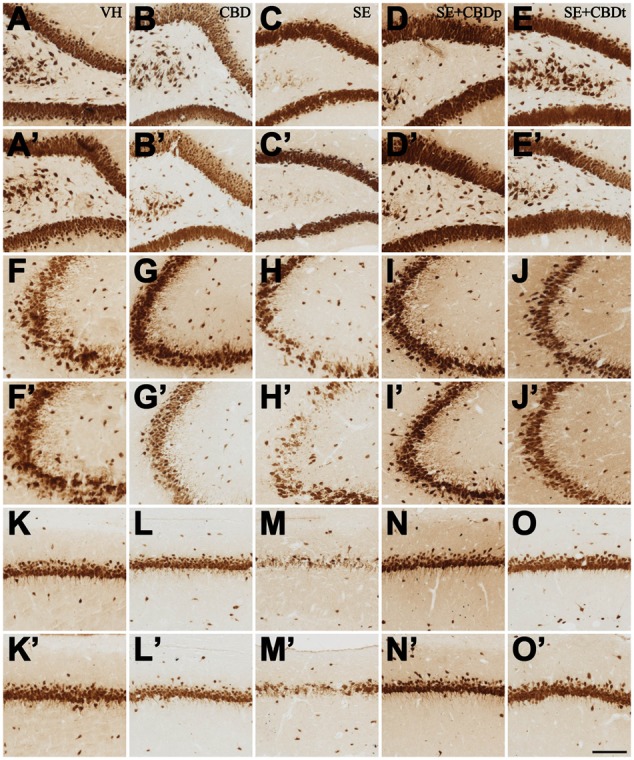
**Representative micrographs from contralateral (A–O)** and ipsilateral **(A’–O’)** hilus **(A–E,A’–E’)**, CA3 **(F–J,F’–J’)**, and CA1 **(K–O,K’–O’)** of VH **(A,A’,F,F’,K,K’)**, CBD **(B,B’,G,G’,L,L’)**, SE **(C,C’,H,H’,M,M’)**, SE+CBDp **(D,D’,I,I’,N,N’)**, and SE+CBDt **(E,E’,J,J’,O,O’)** rats 3 days after SE, submitted to NeuN immunohistochemistry. See the intense neuron loss throughout the hippocampal subfields of a SE rat, both ipsilateral and contralateral to pilocarpine injection, compared to VH and CBD controls. The neuroprotective effect of CBD on SE-induced neuron loss is visible in the SE+CBD groups, as statistically confirmed in **Figure 8**. HIL, hilus; IPSI, ipsilateral side to pilocarpine injection; CONTRA, contralateral side to pilocarpine injection. The bar in **O’** indicates 100 μm.

The 3 days post-SE histological evaluation showed lower neuron density in the SE group, compared to the controls (VH and CBD), in contralateral granule cell layer and in contralateral and ipsilateral, hilus, CA3, and CA1 (ANOVA, *p* < 0.01; **Figures 8A,B**, **9**). SE had also lower neuron density than CBD in the ipsilateral granule cell layer (ANOVA, *p* = 0.042). SE+CBDp, and SE+CBDt groups had higher neuron density than SE group in the contralateral and ipsilateral hilus, CA3, and CA1 (ANOVA, *p* ≤ 0.04). In the contralateral hippocampus (**Figure 8A**), SE+CBDt had lower neuron density than CBD in the granule cell layer and CA1 (ANOVA, *p* ≤ 0.037), and than SE+CBDp in the granule cell layer (ANOVA, *p* = 0.048). In ipsilateral granule cell layer (**Figure 8B**), SE had lower neuron density than CBD and SE+CBDp (ANOVA, *p* ≤ 0.042) SE+CBDt group had lower neuron density than SE+CBDp (ANOVA, *p* < 0.04). In ipsilateral CA3 (**Figure 8B**), SE+CBDp also had higher neuron density than CBD and SE+CBDt (ANOVA, *p* ≤ 0.038). SE+CBDt had lower neuron density than VH, CBD, and SE+CBDp in ipsilateral CA1 (ANOVA, *p* ≤ 0.04; **Figure 8B**). Our findings indicated that CBD administration, both before (SE+CBDp) and before and after (SE+CBDt) pilocarpine-induced SE protected hippocampal sector from neuron loss up to 3 days after the SE.

## Discussion

In the present study, CBD had protective effects on pilocarpine-induced SE, reducing the number of rats that developed SE following intrahippocampal pilocarpine injection, increasing SE behavioral latency, and reducing SE severity from Racine 4 to Racine 3. CBD also affected contralateral LFP, increasing the latency to epileptiform discharges and reducing the relative powers in delta and theta oscillations. Only in the pretreatment group (SE+CBDp), CBD reduced contralateral relative delta oscillations up to 3 days after SE and increased the exploratory behavior 1 day after SE. Finally, CBD preventive (SE+CBDp) and CBD preventive and therapeutic treatment (SE+CBDt) reduced neuronal degeneration and neuron loss both in the ipsilateral and contralateral hippocampus.

Physiologically, cannabinoids have been suggested to play a major role in tissue homeostasis, modulating neurotransmission in the central nervous system (Marsicano and Lutz, 2006). Endocannabinoids are synthesized in response to hyperexcitability and, acting at presynaptic receptors, modulate the release of neurotransmitters (Lutz, 2004; Marsicano and Lutz, 2006). Similarly to endocannabinoids, CBD can modulate neuronal excitability via CB1-depentent and CB1-independent pathways (Pertwee, 2008). The most relevant effect of CBD on neuronal excitability is the regulation of intracellular Ca^2+^ in hippocampal neurons during hyperexcitability states (Ryan et al., 2009). Additional effects of CBD on neurotransmission are the reduction of N-Methyl-D-Aspartate receptor (NMDAR) NR1 subunit expression (Mao et al., 2015), facilitation of serotoninergic 5-hydroxytryptamine receptor 1a (5-HT1a) dependent transmission (Campos et al., 2012; Jones et al., 2012), and reduction of γ-aminobutyric acid (GABA), serotonin, and norepinephrine uptake by synapses (Banerjee et al., 1975). In line with these actions, studies with chemically induced and electrically induced seizures have shown anticonvulsant effects of CBD (Consroe and Wolkin, 1977; Consroe et al., 1982; Jones et al., 2009, 2012). In our study, CBD injection before pilocarpine-induced SE doubled the latency to SE onset and reduced SE severity and mortality. Moreover, one rat from SE+CBD groups had spontaneous SE remission, which did not occur in the SE group. In addition to the behavioral effects, CBD delayed seizure spread for the contralateral side, increased afterdischarges latency and reduced the relative powers in delta and theta frequencies at the contralateral hippocampus. In TLE patients, regardless of the specific neuropathological changes, most patients present flattening of EEG activity, fast activity associated with a reduction in amplitude as the ictal pattern, followed by increased rhythmic delta and theta activity during seizure development and propagation (Pelliccia et al., 2013). Pathological delta and theta activities are indicators of several neuropathological conditions, such as ischemia, tumors, neurodegenerative diseases, and epilepsy (Gloor et al., 1977; de Jongh et al., 2003; Babiloni et al., 2006; Pelliccia et al., 2013). In an ischemia model, 5-mg/kg CBD injection 5 min after bilateral carotid occlusion prevented the increase in delta frequency seen in the vehicle-treated ischemic gerbils (Braida et al., 2003). However, one study showed that clinical improvement is not always followed by EEG improvement in drug-resistant patients (Cunha et al., 1980). Our results suggest that CBD exerts its therapeutic effect by modulating not only the behavioral characteristics of seizures but also the oscillatory activity in the power spectrum of SE model.

There are limited clinical studies on CBD anticonvulsant properties. A prospective, double-blind, controlled study conducted with 15 drug-resistant TLE patients showed that CBD increased antiepileptic drug efficacy, promoting seizure remission in 50% of cases and reducing seizure frequency in 37.5% of patients (Cunha et al., 1980). Another study showed reduction in seizure frequency after CBD administration (Trembly and Sherman, 1990). In contrast, a randomized study with drug-resistant cases failed to indicate an anticonvulsant effect of CBD (Ames and Cridland, 1986). More recently, an open-label study showed that CBD, co-administered with antiepileptic drugs, reduces seizure frequency in severe drug-resistant epilepsies such as Dravet syndrome (Devinsky et al., 2016). CBD also reduced seizure frequency by half in tuberous sclerosis cases (Hess et al., 2016). These clinical studies, although involving a small number of drug-resistant patients, showed the potential action of CBD as an anticonvulsant, especially if added to the traditional antiepileptic drug therapy. New studies should evaluate the potential benefits of CBD administration during the latent period of TLE. Unlike most epilepsies, patients with TLE often present a history of a seizure in the early childhood, followed by a latent period that culminates with the appearance of spontaneous recurrent seizures later in life (Engel, 1996). This latent period, when several plastic changes take place in the temporal lobe (Pitkanen and Sutula, 2002), is an important therapeutic window to prevent seizure recurrence in TLE. Moreover, as an adjuvant to the already established therapeutic strategies, CBD could also have an important impact in SE management, a medical emergency with high mortality, morbidity, and often associated with significant neuron loss (Neligan and Shorvon, 2009; Trinka et al., 2015; Grover et al., 2016). In light of the protective effects against pathological oscillatory changes and neurodegeneration seen in our study, CBD could improve SE management and latent period treatment of epilepsy patients.

Impaired cognition, lethargy, headaches, and even depression can follow the seizures in drug-resistant epilepsy patients (Fisher and Schachter, 2000). The pilocarpine-induced SE model often presents a post-ictal lethargy state, with a significant decrease in exploratory activity (Turski et al., 1983b; Prut and Belzung, 2003). Evidence of functional recovery promoted by CBD treatment come from ischemia models, where CBD improved the neurological function scores, the motor coordination, and reduced the hyper locomotory behavior up to 3 days after the ischemic brain insult (Braida et al., 2003; Hayakawa et al., 2008). The protection of neurological function seen in ischemia models can be extended to the protective effects described in mesial TLE (MTLE). However, in our study, only the pretreated group (SE+CBDp) had decreased prostration/post-ictal lethargy, as shown by increased vertical exploratory activity. Although SE+CBDp group received only one CBD injection before SE induction, detectable levels of CBD were seen in the plasma up to 3 days post-SE, as shown by the gas chromatography evaluation. The presence of CBD in the single dose group was expected, given that the half-life of CBD elimination has been estimated between 2 and 5 days (Consroe et al., 1991). As for the SE+CBDt group, there was no effect of CBD treatment on delta activity and post-ictal prostration. Since CBD acts with an inverted U-shaped dose-response curve (Guimaraes et al., 1990; Campos et al., 2012; Levin et al., 2014; Nazario et al., 2015), the higher CBD plasma levels of SE+CBDt could be responsible for the lack of CBD effect on exploratory and electrographic activities after SE, if compared to the single treatment group SE+CBDp, which had lower doses of plasma CBD.

Patients and an animal model of MTLE often present neurodegenerative damage in different brain areas (Araujo et al., 2006; Diniz et al., 2011; Kandratavicius et al., 2015; Peixoto-Santos et al., 2015; Wolf et al., 2016). In particular, severe loss of hilar interneurons and pyramidal neurons in CA3 and CA1 is commonly seen in the hippocampus of MTLE patients and animal models (Turski et al., 1983a; Leite et al., 1996; Covolan et al., 2000). The neuroprotective actions of CBD were demonstrated in experimental models of cerebral ischemia, where CBD treatment reduced the infarct size and prevented hippocampal neuron loss, especially in the CA1 and hilus subfields (Braida et al., 2003; Hayakawa et al., 2008; Schiavon et al., 2014). We can transpose these protective effects of CBD in ischemia models for epilepsy since these disorders share common mechanisms of neuronal loss involving Ca^2+^ and excitotoxicity mechanisms (Auer and Siesjo, 1988). In epilepsy, the neuronal loss is a dynamic process that comprises multiple factors. Among these factors, excessive Ca^2+^ influx mediated by voltage-gated and NMDAR activation during prolonged seizures, increased reactive oxygen species production, increased Zn^2+^ accumulation in neurons, amongst others (Choi, 1992; Cole et al., 2000). The elevation of intracellular ions activates several biochemical cascades and proteases, lipases, and endonucleases, which will promote degeneration and cell death (Choi, 1992). Since cannabinoids can regulate intracellular Ca^2+^ homeostasis (Drysdale et al., 2006; Ryan et al., 2009; De Petrocellis et al., 2011), we can speculate that Ca^2+^ mechanisms are crucial for the neuroprotective action of CBD. Other significant neuroprotective effects of CBD are modulation of neurotransmitters’ release, modulation of NMDA receptor activation, and inhibition of reactive astrogliosis (Lutz, 2004; Marsicano and Lutz, 2006; Schiavon et al., 2014). In the present study, CBD administration protected hippocampal neurons from the degeneration and death common to pilocarpine-induced SE.

Some limitations of our study must be addressed. Published data suggest that greater intensity of seizures in the acute phase of SE increased the chance to develop recurrent seizures in the chronic phase (Lemos and Cavalheiro, 1995). Since treatment with CBD attenuated seizure severity during SE, it is expected that CBD treatment attenuates the severity of the chronic phase of the pilocarpine rat model of TLE. Thus, a further study should evaluate the effect of CBD pre-SE treatment in the spontaneous seizure frequency of the pilocarpine-induced SE model. Moreover, future studies should also evaluate if the neuroprotection promoted by this brief CBD treatments would remain in the chronic period without the need of further CBD injections. Finally, an important limitation of our study was the lack of a post-SE CBD treatment only (i.e., without the pre-SE CBD injection). This group would be crucial for evaluation the add-on effect of CBD in the human SE and in the post initial precipitant injury management, as commented above.

## Conclusion

This study showed that CBD treatment reduces the behavioral severity and oscillatory electrographic changes of SE, the post-ictal lethargy, and the neuronal loss associated with the pilocarpine-induced SE rat model. More studies are needed to understand the specific mechanisms of action related to the neuroprotective and anticonvulsant effects of CBD in epilepsy.

## Author Contributions

Conceived and designed the experiments: JL, JH, JC, and RD. Performed the experiments: RD, RS, IE, and JD. Analyzed the data: RD, JP-S, LK, IE, and JD. Contributed reagents/materials/analysis tools: BSD, MA, AZ, JC, JH, and JL. Wrote the paper: RD, JP-S, LK, AZ, JC, JH, and JL.

## Conflict of Interest Statement

JH, AZ, and JC are co-inventors (Mechoulam R, JC, Guimaraes FS, AZ, JH, Breuer A) of the patent “Fluorinated CBD compounds, compositions and uses thereof. Pub. No.: WO/2014/108899. International Application No.: PCT/IL2014/050023”; Def. US no. Reg. 62193296; 29/07/2015; INPI em 19/08/2015 (BR1120150164927). University of São Paulo licensed it to Phytecs Pharm (Resolução USP No. 15.1.130002.1.1). University of São Paulo has an agreement with Prati-Donaduzzi (Toledo, Brazil): “Desenvolvimento de um produto farmacêutico contendo canabidiol sintético e comprovação de sua segurança e eficácia terapêutica na epilepsia, esquizofrenia, doença de Parkinson e transtornos de ansiedade”. JC received a travel support from BSPG-Pharm. The other authors declare that the research was conducted in the absence of any commercial or financial relationships that could be construed as a potential conflict of interest.

## Footnotes

1 http://chronux.org/

## Supplementary Material

FIGURE S1 **Cannabidiol (CBD) plasma levels.** CBD plasma concentration pre- and post-SE induction, measured with liquid phase gas chromatography. At day 0 (pre-SE), all rats injected with CBD (groups CBD, SE+CBDp, and SE+CBDt) had detectable CBD plasma 90 min post infusion. At days 1 and 3, CBD levels decreased in the pretreatment group (SE+CBDp, medium green bar), whereas rats infused with CBD pre-SE and every 12 h post-SE (SE+CBDt, dark green bar) presented with increased CBD levels over time. The light green bar indicates CBD only group, and graphs are expressed as mean ± standard error.

FIGURE S2 **Local field potential (LFP) register equipment.** In **A** is shown a rat attached to the register cable inside the register box, which in turn is attached to an analogic-digital converter connected to a computer **(B)**. Inside the box there is a webcam, a light and a cooler **(A)**.

FIGURE S3 **Representative photo of a rat inside the open field apparatus (A)**. Rat movement is detected by a series of infrared sensors (arrows), being three lower sensors that detect horizontal movement (white arrows) and one high sensor (black arrow) that detects vertical movements (i.e., rises). The apparatus is attached to a computer, and the software provided an image of the horizontal movement of the rats inside the box **(B)** and the place and number of rises **(C)**. The software also provides the distance and velocity of every animal in a.txt file (not shown).
