# Supplementary figures and images for: Protective Effects of Cannabidiol against Seizures and Neuronal Death in a Rat Model of Mesial Temporal Lobe Epilepsy
Source: Front Pharmacol. 2017 Mar 17;8:131. doi: 10.3389/fphar.2017.00131 (PMC5355474; doi:10.3389/fphar.2017.00131)

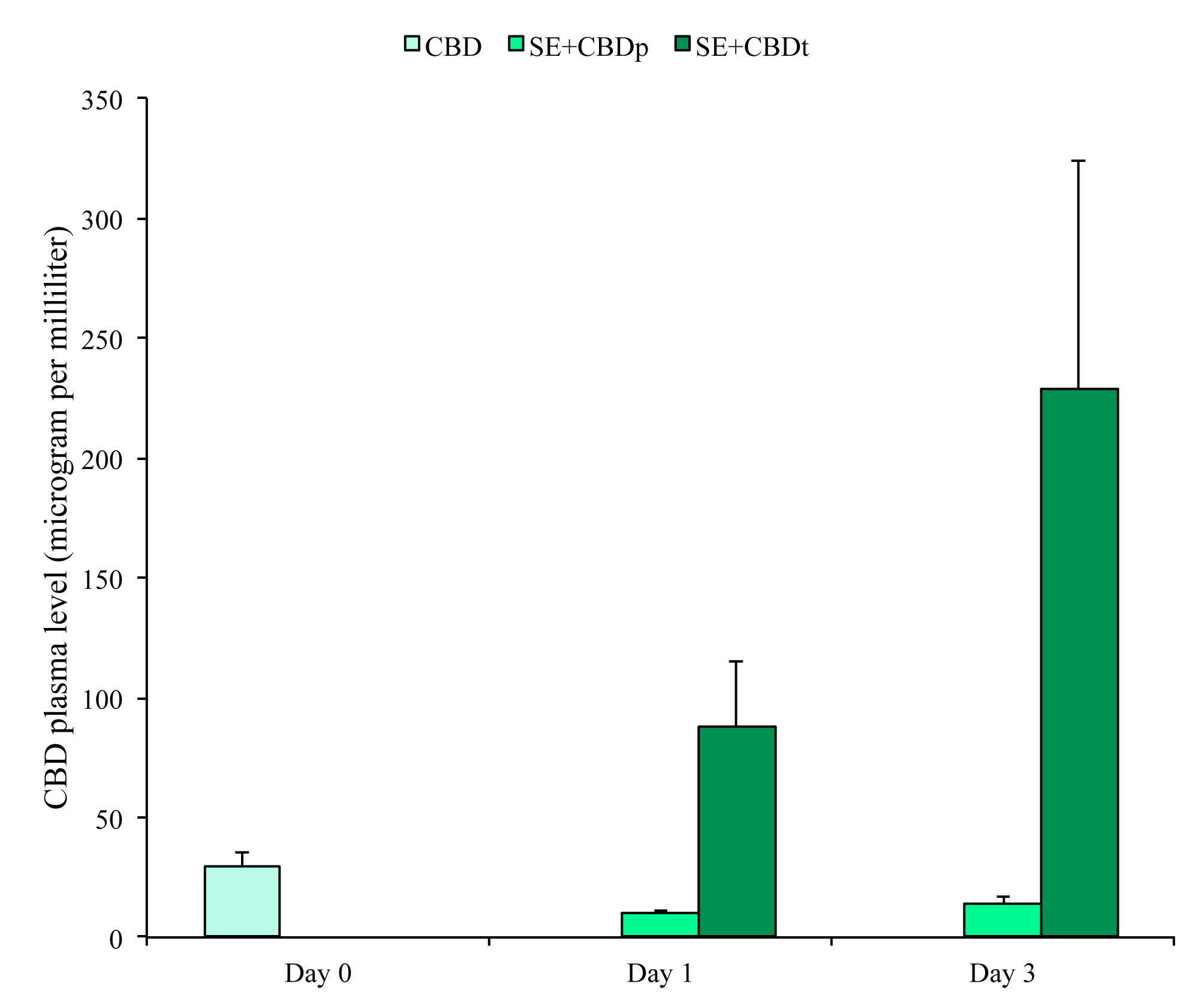

Supplement: FIGURE S1 — Cannabidiol (CBD) plasma levels. CBD plasma concentration pre- and post-SE induction, measured with liquid phase gas chromatography. At day 0 (pre-SE), all rats injected with CBD (groups CBD, SE+CBDp, and SE+CBDt) had detectable CBD plasma 90 min post infusion. At days 1 and 3, CBD levels decreased in the pretreatment group (SE+CBDp, medium green bar), whereas rats infused with CBD pre-SE and every 12 h post-SE (SE+CBDt, dark green bar) presented with increased CBD levels over time. The light green bar indicates CBD only group, and graphs are expressed as mean ± standard error. [file Image_1.TIF]

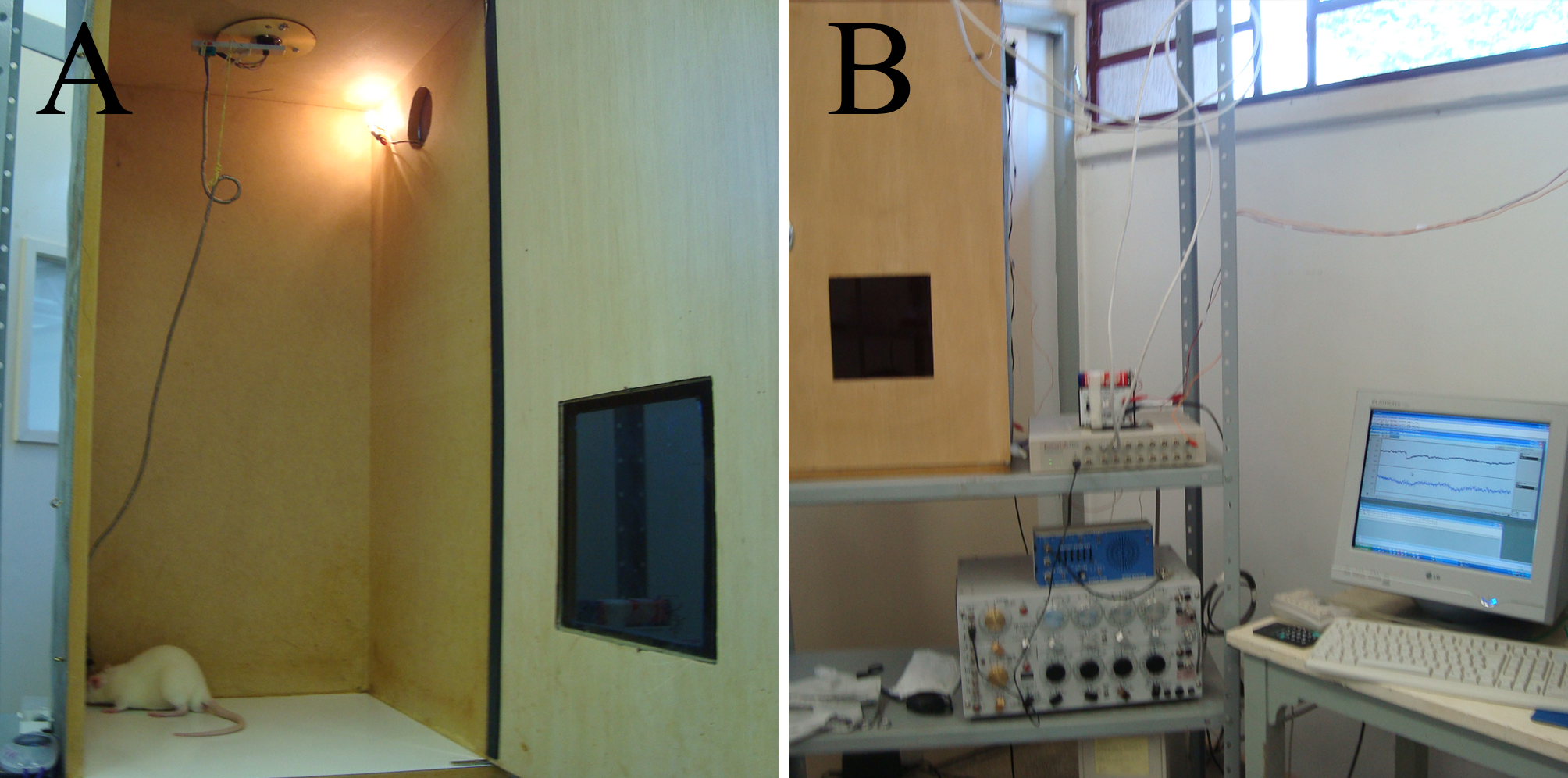

Supplement: FIGURE S2 — Local field potential (LFP) register equipment. In A is shown a rat attached to the register cable inside the register box, which in turn is attached to an analogic-digital converter connected to a computer (B). Inside the box there is a webcam, a light and a cooler (A). [file Image_2.TIF]

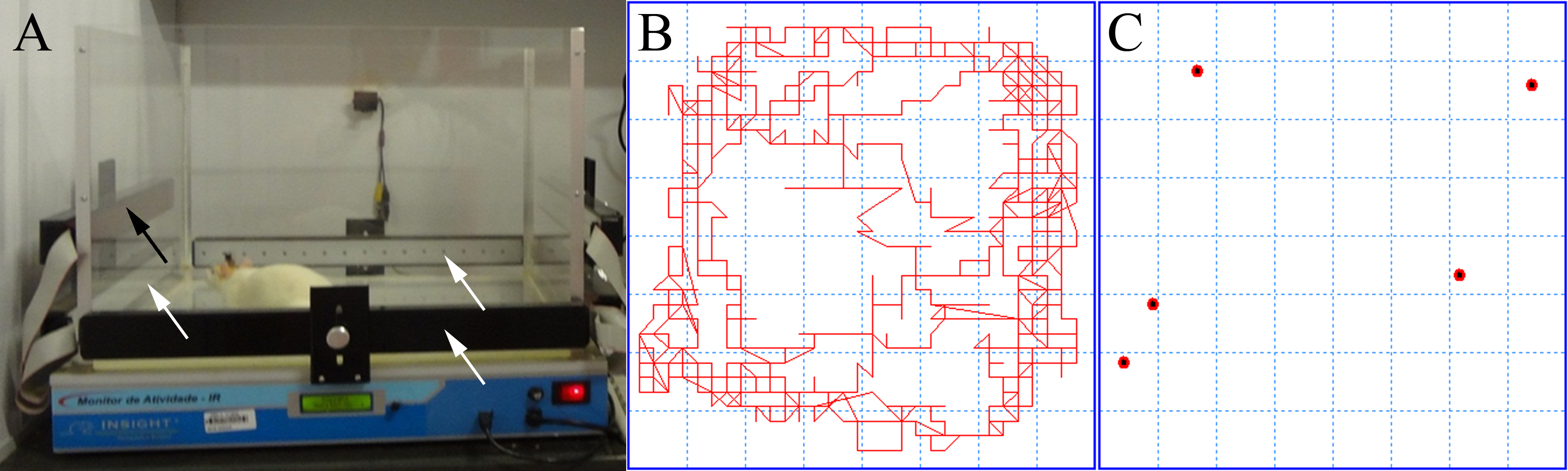

Supplement: FIGURE S3 — Representative photo of a rat inside the open field apparatus (A). Rat movement is detected by a series of infrared sensors (arrows), being three lower sensors that detect horizontal movement (white arrows) and one high sensor (black arrow) that detects vertical movements (i.e., rises). The apparatus is attached to a computer, and the software provided an image of the horizontal movement of the rats inside the box (B) and the place and number of rises (C). The software also provides the distance and velocity of every animal in a.txt file (not shown). [file Image_3.TIF]
